# Muriate of Cocaine

**Published:** 1885-04

**Authors:** W. D. Miller

**Affiliations:** Berlin


### MURIATE OF COCAINE.

BY DR. W. D. MILLER, BERLIN.

I have been using this substance now for some months, and my experience has been such that I was not a little surprised when I read the reports of the wonderful effects produced by the application of a two per cent. solution. I began my experiments with a four per cent. solution, which in the great majority of cases I found so ineffective that I rapidly increased the strength, now employing nothing under a thirty per cent. solution.

It has also been used to a considerable extent at the Dental Institute, in the same strength; and in this strength, in some cases, not in all, it works excellently as an obtunder of sensitive dentine. It may also be used to advantage in performing operations upon the dental pulp, and for anæsthetizing the sometimes extremely sensitive and painful gum before applying the rubber dam; here I have found its effect most grateful.

It is, however, not the object of this letter to present my views on cocaine muriate, but rather those of Dr. Sachs, of Breslau, which you will find in the accompanying letter. On the whole, I regard cocaine muriate as a valuable addition to the dental pharmacopœia, but by no means one of unlimited value.

Dr. Sachs writes as follows: "I first tried a two per cent. solution to relieve the pain in the sensitive dentine, but without success. I then used a ten per cent. solution, which in certain cases worked extremely well. For so-called sensitive dentine this solution is not very efficient, as it cannot act on the pulp when there is a thick layer of dentine between. But in those cases where the cavity is very large and the pulp nearly exposed, the pain of excavating is almost entirely done away with. The success is particularly apparent in very soft, chalky teeth. I had lately to excavate a first lower molar for a Miss of twelve years; the tooth was so sensitive to the instrument that the patient would not allow me to continue. I used a ten per cent. solution of cocaine, and after about one minute the sensitiveness was so far reduced that I could easily remove the great thick masses of softened dentine with a spoon-shaped excavator; but, unfortunately, the insensibility was so complete that in my joy over the success I worked too energetically, and exposed the pulp; but even this the patient did not notice.

"Since then I have often used cocaine, and in very many cases with brilliant success, so that I consider this medicine a most valuable contribution to our materia medica.

"I have used it, also, with the greatest satisfaction in cases where the sensitiveness of the soft palate is so great as to render the operation of taking an impression exceedingly disagreeable, if not impossible. I paint the whole soft palate and the surrounding soft parts with a two per cent. solution, and am able, after about one minute, to take a good impression without the least difficulty.

"In excavating, I use the material in the following manner: First, I adjust the rubber dam and dry the cavity, dip a pellet of cotton into the solution, and leave it for about a minute in the tooth; then remove it and again dry the cavity thoroughly, using the hot air syringe, and then begin to excavate. If the solution remains in the tooth too long, the anæsthetic effect is almost or quite lost."

